# Antarctic Starfish (Echinodermata, Asteroidea) from the ANDEEP3 expedition

**DOI:** 10.3897/zookeys.185.3078

**Published:** 2012-04-23

**Authors:** Bruno Danis, Michel Jangoux, Jennifer Wilmes

**Affiliations:** 1 ANTABIF, 29, rue Vautier, 1000, Brussels, Belgium; 2 Université Libre de Bruxelles, 50, av FD Roosevelt, 1050, Brussels, Belgium

**Keywords:** Asteroidea, Biodiversity, Deep-Sea, Census of Antarctic Marine Life, Census of Marine Life, ANDEEP cruises, Polarstern, Sea-stars, Starfish

## Abstract

This dataset includes information on sea stars collected during the ANDEEP3 expedition, which took place in 2005. The expedition focused on deep-sea stations in the Powell Basin and Weddell Sea.

Sea stars were collected using an Agassiz trawl (3m, mesh-size 500µm), deployed in 16 stations during the ANTXXII/3 (ANDEEP3, PS72) expedition of the RV Polarstern. Sampling depth ranged from 1047 to 4931m. Trawling distance ranged from 731 to 3841m. The sampling area ranges from -41°S to -71°S (latitude) and from 0 to -65°W (longitude). A complete list of stations is available from the PANGAEA data system (http://www.pangaea.de/PHP/CruiseReports.php?b=Polarstern), including a cruise report (http://epic-reports.awi.de/3694/1/PE_72.pdf).

The dataset includes 50 records, with individual counts ranging from 1-10, reaching a total of 132 specimens.

The andeep3-Asteroidea is a unique dataset as it covers an under-explored region of the Southern Ocean, and that very little information was available regarding Antarctic deep-sea starfish. Before this study, most of the information available focused on starfish from shallower depths than 1000m. This dataset allowed to make unique observations, such as the fact that some species were only present at very high depths (*Hymenaster crucifer*, *Hymenaster pellucidus*, *Hymenaster praecoquis*, *Psilaster charcoti*, *Freyella attenuata*, *Freyastera tuberculata*, *Styrachaster chuni* and *Vemaster sudatlanticus* were all found below -3770m), while others displayed remarkable eurybathy, with very high depths amplitudes (*Bathybiaster loripes* (4842m), *Lysasterias adeliae* (4832m), *Lophaster stellans* (4752m), *Cheiraster planeta* (4708m), *Eremicaster crassus* (4626m), *Lophaster gaini* (4560m) and *Ctenodiscus australis* (4489m)).

Even if the number of records is relatively small, the data bring many new insights on the taxonomic, bathymetric and geographic distributions of Southern starfish, covering a very large sampling zone. The dataset also brings to light six species, newly reported in the Southern Ocean.

The quality of the data was controlled very thoroughly, by means of on-board Polarstern GPS systems, checking of identification by a renowned specialist (Prof. Michel Jangoux, Université Libre de Bruxelles), and matching to the Register of Antarctic Marine Species (RAMS) and World Register of Marine Species (WoRMS). The data is therefore fit for completing checklists, for inclusion in biodiversity patterns analysis, or niche modeling. It also nicely fills an information gap regarding deep-sea starfish from the Southern Ocean, for which data is very scarce at this time. The authors may be contacted if any additional information is needed before carrying out detailed biodiversity or biogeographic studies.

## Project details

**Project title:** Bianzo - Biodiversity of Antarctic Zoobenthos

**Personnel:** Bruno Danis

**Funding:** Belgian Science Policy Office (BELSPO, cash), Alfred Wegener Institute (AWI, in kind), Marine Biology Lab (ULB, in kind)

**Study area descriptions/descriptor:** The study area of this dataset was set in the Southern Ocean and focused on deep sea stations distributed on the continental slopes of the eastern Weddell Sea (off Kapp Norvegia) and western Weddell Sea and the South Orkney Islands, and deep Cape, Agulhas, Weddell and Powell Basins Southern Ocean. The Southern Ocean deep-sea is a very under sampled area, according to a recent gap analysis carried out by Griffiths et al. (2010).

**Design description:** BIANZO (Biodiversity of Antarctic Zoobenthos) investigated biodiversity patterns of the Antarctic zoobenthos and their causal processes for three representative groups of different size categories: nematodes (meiobenthos), amphipods (macrobenthos) and echinoids (megabenthos). Trophodynamic aspects of these benthic groups and their ability to cope with temperature and temperature-related changes (food composition and availability, pH of the seawater...) will be studied mainly in an experimental approach. Information collected in previous studies and in the first two work packages will be used to initiate the development of a model about the possible changes in the benthic communities due to global environmental change. BIANZO generated the initial core data system upon which SCAR’s Marine Biodiversity Information Network (SCAR-MarBIN) was built. As SCAR-MarBIN is the Antarctic Node of the international OBIS network,the BIANZO data system was designed to comply with the OBIS standards. BIANZO served as a model to give a single access to three heterogeneous databases, each focusing on the specific groups of interest (Amphipoda, Nematoda, Echinoidea).

Regarding the dataset, the existing Data Toolkit from SCAR-MarBIN was used (http://www.scarmarbin.be/documents/SM-FATv1.zip), following the OBIS schema (http://iobis.org/data/schema-and-metadata). The dataset was uploaded in the ANTOBIS database (the geospatial component of SCAR-MarBIN), and the taxonomy was matched against the Register of Antarctic Marine Species, using the Taxon Match tool (http://www.scarmarbin.be/rams.php?p=match)

## Taxonomic coverage

**General taxonomic coverage description:** This dataset focuses on Starfish (Echinodermata: Asteroidea). It includes data on 6 orders (Forcipulatida, Notomyotida, Paxillosida, Spinulosida, Valvatida, Velatida) and 11 families (Asteriidae, Astropectinidae,Benthopectinidae, Echinasteridae, Freyellidae, Goniasteridae, Labidiasteridae, Notasteriinae, Porcellanasteridae, Pterasteridae, Solasteridae). The most represented families are the Astropectinidae (Paxillosida), followed by Porcellanasteridae (Paxillosida) and Pterasteridae (Velatida).

### Taxonomic ranks

**Order:** Forcipulatida, Notomyotida, Paxillosida, Spinulosida, Valvatida, Velatida

**Family:** Asteriidae, Astropectinidae, Benthopectinidae, Echinasteridae, Freyellidae, Goniasteridae, Labidiasteridae, Notasteriinae, Porcellanasteridae, Pterasteridae, Solasteridae

**Genus:** *Freyella*, *Bathibiaster*, *Cheiraster*, *Diplasterias*, *Dytaster*, *Eremicaster*, *Freyastera*, *Freyella*, *Hymenaster*, *Hyphalaster*, *Lophaster*, *Lysasterias*, *Marsipaster*, *Notasterias*, *Notioceramus*, *Parachaster*, *Psalidaster*, *Psilaster*, *Rhopiella*, *Ripaster*, *Styracaster*

**Species:** *Dytaster felix*, *Freyella attenuata*, *Psalidaster mordax*, *Lophaster gaini*, *Bathybiaster loripes*, *Psilaster charcoti*, *Pteraster hirsutus*, *Freyastera tuberculata*, *Hymenaster pellucidus*, *Hymenaster praecoquis*, *Benthopecten pedicifer*, *Hyphalaster inermis*, *Eremicaster pacificus*, *Lophaster stellans*, *Eremicaster crassus*, *Rhopiella hirsuta*, *Notioceramus anomalus*, *Hymenaster crucifer*, *Styracaster chuni*, *Cheiraster planeta*, *Pteraster spinosissimus*, *Hyphalaster scotiae*, *Notasterias pedicellaris*, *Diplasterias brucei*, *Lysasterias adeliae*

## Spatial coverage

**General spatial coverage:** ANDEEP 3 cruise track, from Cape Town (SA) to Punta Arenas (CH). Four study regions were selected, but the main focus was on the Powell Basin and the Weddell Basin of the Weddell Sea, and their slopes. Two comparative samples were taken further north in the adjacent Agulhas and southern Cape Basins, which are separated from each other by the Agulhas Ridge. Four study regions were selected, but the main focus was on the Powell Basin and the Weddell Basin of the Weddell Sea, and their slopes. The major South Atlantic deep-sea basins started forming during Jurassic and Cretaceous times in connection with the Gondwana break-up and seafloor spreading (Brandt et al. 2007; Lawver and Gahagan 2003). The Weddell Basin is separated from the northerly basins by the South- west India Ridge (LaBrecque 1986). The Powell Basin on the western side of the Weddell Sea was formed in the Tertiary by geological processes opening the Drake Passage and tectonic movements in the Scotia Sea (Lawver and Gahagan 2003; Mitchell et al. 2000). The oceanography of the deep South Atlantic seafloor is defined by its prominent water mass, the Antarctic Bottom Water (Tomczak and Godfrey 2001). The Antarctic Bottom Water expands north- wards into the Atlantic basins east and west of the Mid-Atlantic Ridge, like the Agulhas Basin, but can only enter the basins north of the Walvis Ridge (e.g., Cape Basin) via the northerly Romanche Fracture Zone. The Weddell Sea Bottom Water (WSBW), defined by a temperature of 0.7 1C and a salinity of 34.64 ppt (Orsi et al. 1993), is the main water mass above the Weddell Sea benthos (Fahrbach et al. 2001). The WSBW flows from the western Weddell Sea into the Scotia Sea and South Sandwich Forearc, and its circulation is driven by the Weddell Sea gyre. The sediments in the bathyal and abyssal Weddell and Powell Basins are dominated by silt and clay.

**Coordinates:** 71°18'36"S and 61°30'0"S Latitude; 64°38'24"W and 0°0'0"E Longitude

**Temporal coverage:** January 26, 2005 – March 30, 2005

**Natural collections description**

**Parent collection identifier:** Marine Biology Lab, Free University of Brussels. Antarctic Echinoderms Collection

**Collection name:** ANDEEP3 Seastars

**Collection identifier:** Michel Jangoux

**Formation period:** January to March 2005

**Specimen preservation method:** Alcohol

## Methods

**Method step description:** see quality control above.

**Study extent description:** Four study regions were selected, but the main focus was on the Powell Basin and the Weddell Basin of the Weddell Sea, and their slopes. Two comparative samples were taken further north in the adjacent Agulhas and southern Cape Basins, which are separated from each other by the Agulhas Ridge.

**Sampling description:** A 3-m wide Agassiz trawl (AGT) was deployed at two locations in the South Atlantic and 14 locations in the Southern Ocean during the PFS Polarstern expedition ANT XXII/3 WECCON 2005— ANDEEP III in January–April 2005. The sample depths ranged from 1047 to 4931 m, sampling continental slopes of the eastern Weddell Sea (off Kapp Norvegia) and western Weddell Sea and the South Orkney Islands, and deep Cape, Agulhas, Weddell and Powell Basins. At the stations 074-7, 078-11 and 081-9, the cod end mesh size was 10mm, while at all other stations, an inlet of 500 mm mesh size was inserted. The 500 mm mesh size was used because of smaller adult size of deep- sea macrobenthos compared to shelf macrobenthos. The deployment protocol was standardised to 10 min trawling at 1 knot with 1.5× cable length to water depth to facilitate comparability between the different sites. At station 059-10, the AGT was trawled for 20 min. The haul distances were calculated from the time the Agassiz trawl travelled on the ground. The tension meter of the winch clearly indicated when the AGT left the seabed. Haul length varied from 731 to 3841m. Sample volumes were estimated and the general sediment composition was noted. Mega- and larger macrofauna were separated by eye on deck. The taxa of each trawl sample were identified to morphospecies level.

**Quality control description:** The initial geo-referencing was done by means of the RV Polarstern onboard GPS systems. Geospatial data was directly imported from those systems to avoid potential errors in transcribing. Samples identification was supervised and checked by Michel Jangoux, Marine Biology Lab, Université Libre de Bruxelles. The taxonomic names were matched against two authoritative, expert-driven species registers: the Register of Antarctic Marine Species (RAMS) and the World Register of Marine Species (WoRMS). The automatic matching tools available on both these web sites were utilized.

## Data resources

The data underpinning the analyses reported in this paper are deposited at GBIF, the Global Biodiversity Information Facility, http://ipt.biodiversity.aq/resource.do?r=andeep3_asteroidea.

## Datasets

### Dataset description

There is no dataset published through Darwin Core Archive format for this resource. Currently described datasets are listed in the section External datasets.

**Language:** English

**Licenses of use:** This work is licensed under a Creative Commons CCZero 1.0 License http://creativecommons.org/publicdomain/zero/1.0/legalcode

### External datasets

**Dataset description**

**Object name:** SCAR-MarBIN DiGIR Server

**Format name:** OBIS schema

**Format version:** v1.1

**Distribution:** http://w2.scarmarbin.be/digir2/digir.php

**Dataset description**

**Object name:** GBIF data portal

**Format name:** DarwinCore

**Distribution:** http://data.gbif.org/datasets/resource/7928/

**Metadata language:** English

**Date of metadata creation:** 2011-12-03

**Hierarchy level:** Dataset
